# Chromosome genome assembly of the *Camphora longepaniculata* (Gamble) with PacBio and Hi-C sequencing data

**DOI:** 10.3389/fpls.2024.1372127

**Published:** 2024-06-26

**Authors:** Kuan Yan, Hui Zhu, Guiling Cao, Lina Meng, Junqiang Li, Jian Zhang, Sicen Liu, Yujie Wang, Ruizhang Feng, Salma A. Soaud, Mohamed A. Abd Elhamid, Rania M. Y. Heakel, Qin Wei, Ahmed H. El-Sappah, Dafu Ru

**Affiliations:** ^1^ Faculty of Agriculture, Forestry and Food Engineering, Yibin University, Yibin, China; ^2^ Sichuan Oil Cinnamon Engineering Technology Research Center, Yibin University, Yibin, China; ^3^ State Key Laboratory of Herbage Improvement and Grassland Agro-Ecosystem, College of Ecology, Lanzhou University, Lanzhou, China; ^4^ Genetics Department, Faculty of Agriculture, Zagazig University, Zagazig, Egypt

**Keywords:** *Camphora longepaniculata*, high-throughput sequencing, protein-coding genes, traditional Chinese medicine, terpenoid

## Abstract

**Introduction:**

*Camphora longepaniculata*, a crucial commercial crop and a fundamental component of traditional Chinese medicine, is renowned for its abundant production of volatile terpenoids. However, the lack of available genomic information has hindered pertinent research efforts in the past.

**Methods:**

To bridge this gap, the present study aimed to use PacBio HiFi, short-read, and highthroughput chromosome conformation capture sequencing to construct a chromosome-level assembly of the *C. longepaniculata* genome.

**Results and discussion:**

With twelve chromosomes accounting for 99.82% (766.69 Mb) of the final genome assembly, which covered 768.10 Mb, it was very complete. Remarkably, the assembly’s contig and scaffold N50 values are exceptional as well—41.12 and 63.78 Mb, respectively—highlighting its excellent quality and intact structure. Furthermore, a total of 39,173 protein-coding genes were predicted, with 38,766 (98.96%) of them being functionally annotated. The completeness of the genome was confirmed by the Benchmarking Universal Single-Copy Ortholog evaluation, which revealed 99.01% of highly conserved plant genes. As the first comprehensive assembly of the *C. longepaniculata* genome, it provides a crucial starting point for deciphering the complex pathways involved in terpenoid production. Furthermore, this excellent genome serves as a vital resource for upcoming research on the breeding and genetics of *C. longepaniculata*.

## Introduction

**Figure 1:**
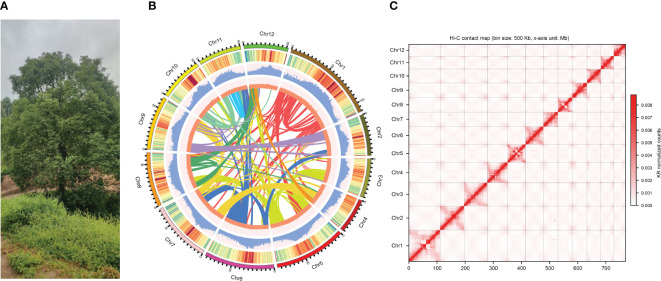
Sampling and genome assembly of *Camphora longepaniculata*. **(A)** The sequenced individual of *C. longepaniculata* leaves from Chinese province of Sichuan (27°50’ N, 105°20’ E). **(B)** Genome features from the *C. longepaniculata* assembly. From outer to inner: (1) genome chromosomes, (2) gene density, (3) repeat density, (4) GC (guanine-cytosine) content, and (5) synteny information. **(C)** Genome-wide chromosomal contact map of *Camphora longepaniculata* using Hi-C data. Interaction frequency distribution of Hi-C links among chromosomes shows in color key of heatmap ranging from white to dark red indicating the frequency of Hi-C interaction links from low to high.

A species of evergreen tree native to southwestern China, namely the Yibin region of Sichuan, *Camphora longepaniculata* (Gamble) Y. Yang, Bing Liu, and Zhi Yang, is important both culturally and industrially (**Figure 1A**; Wu et al., 2022). Because of its fragrant qualities, it has become a traditional plant of interest that is both extensively grown and highly valued in regional customs. Its potential to extract essential oils from a variety of parts, including roots, stems, leaves, and seeds, accounts for most of its industrial value. Notably, the bulk is made up of leaf essential oil, which has a high concentration of terpenoids (>85%) (Hu et al., 2012). Prior research has revealed 1,8-cineole, α-terpilenol, and γ-terpinen as important components of leaf essential oil (Li et al., 2012, 2014). These essential oils are prized for their outstanding antibacterial, anti-inflammatory, and antioxidant qualities (Boutanaev et al., 2015; Wei et al., 2016). As with the biological traits of *C. longepaniculata*, studies have been conducted to investigate the molecular underpinnings of monoterpene production. Previous research has discovered important genes linked to terpene production using transcriptome sequencing technologies (Yan et al., 2017). In addition, Yan et al. (2019) investigated the differential expression of *C. longepaniculata* genes induced by endophytic fungi, revealing significant regulation within the monoterpene synthesis pathway. However, a high-quality genome is required as the starting point for additional research in order to fully comprehend the fundamental molecular mechanisms driving synthesis and oil production.

The genomic terrain of *C. longepaniculata* is still unknown, despite advancements in genomic investigation within the Lauraceae family (Huang et al., 2016; Chaw et al., 2019; Song et al., 2019). Comprehensive and methodical study has been hampered by the lack of complete reference genomic material, particularly with regard to the unclear molecular mechanism of 1,8-cineole production. The goal of this work is to build a chromosome-level genome assembly of the *C. longepaniculata* by utilizing the quick development of high-throughput sequencing technologies including PacBio HiFi, short-read, and high-throughput chromosomal conformation capture (Hi-C) sequencing techniques. A wealth of genetic resources will be made available by this excellent genome assembly, allowing researchers to investigate the evolutionary background, gene functions, and regulatory mechanisms behind *C. longepaniculata*’s growth, development, and monoterpene synthesis. It will also provide a strong basis for the cultivation of high-yielding, high-quality cultivars of *C. longepaniculata*.

## Materials and methods

### Sample collection, DNA extraction, and sequencing

*C. longepaniculata* leaves were obtained in the Chinese province of Sichuan (27°50’ N, 105°20’ E). Genomic DNA was then extracted and processed according to the recommended library preparation methodology by PacBio. Subsequently, the DNA was utilized to construct PacBio sequencing libraries using the standard single molecular real-time bell construction protocol provided by PacBio. The libraries were then sequenced on the PacBio CCS platform. Adapter sequences and short, low-quality reads were removed from the PacBio HiFi reads using SMRTLink (parameters: –min-passes = 3 –min-rq = 0.99). Using the Agencourt AMPure XP-Medium Kit, the same DNA was used to create an Illumina paired-end library with insert sizes ranging from 200 to 400 bp. The Illumina NovaSeq 6000 platform was then used to sequence the resulting library. On the MGISEQ2000 platform, a Hi-C library was sequenced in accordance with the proximo Hi-C plant methodology (https://info.phasegenomics.com/protocols) developed by Phase Genomics (Seattle, WA, United States). After removing adaptors and duplicate reads, raw reads were filtered with a sequencing quality of >Q30 (**Supplementary Table S1**).

Lastly, RNA sequencing was performed on a leaf sample obtained from the same individual. Using the Qiagen RNeasy Plant Mini kit, total RNA was extracted from each sample. Then, with the TruSeq RNA Library Preparation kit, RNA-seq libraries were created, and 11.74 Gb of RNA-seq sequences were obtained by sequencing on the Illumina NovaSeq 6000 platform (Belton et al., 2012) (**Supplementary Table S1**).

### Genome assembly and assessment

Utilizing FASTP v0.20.0 (Chen et al., 2018), the short-read sequences were filtered to exclude adaptor contamination and low-quality reads. Subsequently, genome size, repeat content, and heterozygosity rate were estimated using GCE v1.0.2 tool from 19-mer histograms (Liu et al., 2013).

With the parameters “‘–write-ec –write-paf -u -l 0’’, the PacBio HiFi reads were assembled using Hifiasm v.0.15 (Cheng et al., 2021) and redundant sequences were removed using Purge Haplotigs (Roach et al., 2018). Hi-C data was used to anchor the contigs to the chromosomes. In summary, genuine interaction pairs were obtained by filtering the Hi-C reads using FASTP v0.20.0 (**Supplementary Table S5**; Chen et al., 2018) and selecting unique mapped read pairs using the HiCUP v0.8.0 (Wingett et al., 2015) pipeline. Next, a chromosome-level assembly was created using 3D-DNA v201013 (https://github.com/aidenlab/3d-dna), after the sequence had been aligned against the draft genome assembly using Juicer (Dudchenko et al., 2017). The “embryophyta_odb10” ortholog set was used to assess the quality of the genome using BUSCO v4.05 (Simão et al., 2015).

To ensure the integrity of the *C. longepaniculata* genome assembly, high-quality short-read sequences were mapped back to the genome using the BWA v0.7.17 (Li and Durbin, 2010). This process helps to verify the correct alignment and orientation of the assembled sequences. Following the mapping, duplicate reads were removed using Picard v2.23.6 (broadinstitute.github.io/picard) to prevent redundancy that could skew variant identification results. Subsequently, SNPs (Single Nucleotide Polymorphisms) and InDels (insertions and deletions) were identified using SAMtools v1.10 (Li et al., 2009), which provides insights into potential genomic variations and polymorphisms.

In addition to the above steps, HiFi reads were mapped back to the genome using Minimap2 (Li, 2018). This step further ensures that the high-fidelity, long-read sequencing data aligns accurately with the assembled genome, confirming the assembly’s overall quality and completeness.

### Repeat sequence annotation

Repetitive sequences in the *de novo* repeat library were predicted using RepeatMasker v4.0.7 (Tarailo-Graovac and Chen, 2009), and a *de novo* repeat library was created using RepeatModeler (Flynn et al., 2020) and LTRfinder v1.07 (Xu and Wang, 2007) with default settings. In parallel, homologous repeat prediction was carried out using the Repbase v21.12 database and RepeatMasker v4.0.7 and RepeatProteinMasker v4.0.7 (http://www.repeatmasker.org/cgi-bin/RepeatProteinMaskRequest) (Bao et al., 2015). To create nonredundant repeated sequences, the two sets of anticipated repetitions were concatenated. With Tandem Repeat Finder v4.09 (Benson, 1999), tandem repetitions were found.

Gene prediction in the repeat-masked genome was done using three different methods: homology searching, reference-guided transcriptome assembly, and ab initio prediction. According to Keilwagen et al. (2016), homologous peptides from related species (*Cinnamomum kanehirae*, *Phoebe bournei*, *Litsea cubeba*, *Phoebe kanehirae*, and *Phoebe zhennan*) were aligned with the assembly using GeMoMa v1.6.1 (Keilwagen et al., 2016). Gene structure information was then collected for homolog prediction. Filtered mRNA-seq data were used to align STAR v2.7.3a (Dobin et al., 2013) to the reference genome in order to perform RNA-seq-based gene prediction (Dobin et al., 2013). PASA v2.3.3 (Haas et al., 2008) was used to predict open reading frames (ORFs), and StringTie v1.3.4d (Pertea et al., 2015) was used to assemble the transcripts. A training set was also produced for the *de novo* prediction. The training data was then used for ab initio gene prediction using Augustus v3.3.1 (Stanke et al., 2008) with default settings. After creating an integrated gene collection with EvidenceModeler (EVM, v1.1.1; Haas et al., 2008), miscoded genes were further filtered and genes containing TEs were eliminated using the TransposonPSI program (http://transposonpsi.sourceforge.net/) (Urasaki et al., 2017). PASA was used to identify alternative splicing regions and untranslated regions (UTRs) based on RNA-seq assemblies. For each locus, we kept the longest transcripts, and areas outside of the ORFs were called UTRs. By examining five protein/function databases, the projected protein-coding genes’ domains, motifs, and gene function information were found. Predicted protein-coding genes were thoroughly annotated using InterProScan v5.36 (Zdobnov and Apweiler, 2001), which included predicted signal peptides, transmembrane topologies, functional classifications, GO keywords, protein motifs and domains, and protein family identification. KO words were found using KaaS (https://www.genome.jp/kegg/kaas/) searching the KEGG database. A 1e-5 E value limit was utilized for searches against the Swiss-Prot (Bairoch and Apweiler, 2000), NR (Marchler-Bauer et al., 2011), TrEMBL (Bairoch and Apweiler, 2000), and COG databases (Galperin et al., 2015) using BLASTP v.2.7.1 (Altschul et al., 1997). From these database searches, the top hits were combined to get the final results.

### Annotation of noncoding RNAs

Database searches and model-based prediction were the two methods employed to find noncoding RNA sequences in the genome. With eukaryote parameters, tRNAscan-SE v2.0.12 (Lowe and Eddy, 1997) was used to predict tRNAs. INFERNAL v1.1.5 (Nawrocki and Eddy, 2013) was utilized to search the Rfam database and identify sequences of microRNA, rRNA, small nuclear RNA, and small nucleolar RNA. RNAmmer v1.2 (Lagesen et al., 2007) was used to forecast the rRNAs and their components.

### Orthogroup and functional enrichment analysis

In order to investigate the evolutionary background of *C. longepaniculata*, we obtained the genomes of eleven different species from publicly accessible databases. These species include *Aquilegia coerulea*, *Arabidopsis thaliana*, *Amborella trichopoda*, *Liriodendron chinense*, *Nymphaea colorata*, *Piper nigrum*, *Cinnamomum kanehirae*, *Persea americana*, *Chimonanthus salicifolius*, and *Litsea cubeba* (**Supplementary Table S2**). Furthermore, genes with glaring mistakes were eliminated and the longest transcripts were selected as samples for genes having alternative splice variants. The homology matrix of the orthogroups (gene families) among these chosen species was also inferred using OrthoFinder v2.5.4 (Emms and Kelly, 2019). For the purpose of creating phylogenetic trees using the maximum-likelihood (ML) approach, 230 single-copy gene groupings were found. The single-copy gene groups were subjected to multiple sequence alignment using MAFFT v.7.453 (Katoh and Standley, 2013). Subsequently, the coding sequences (CDS sequences) were aligned using PAL2NAL v.14 (Suyama et al., 2006) in accordance with the alignments of the respective proteins. Each single-copy gene group was given a maximum likelihood phylogenetic tree using IQ-TREE v.2.1.4-beta (Nguyen et al., 2015). Ultimately, these 230 single-copy gene trees were combined using the ASTRAL v.5.6.3 (Zhang et al., 2022) software to create a species tree based on the multispecies coalescent model. We employed a Bayesian relaxed molecular clock technique to estimate the divergence periods between species using the MCMCTree tool in the PAML v.4.9 (Yang, 2007) package. Furthermore, we acquired four fossil constraints for divergence time calibration from the TimeTree website (http://www.timetree.org): Between *A. trichopoda* and *N. colorata*, 179–205 Mya; between A. coerulea and P. nigrum, 151–170 Mya; between *A. coerulea* and *A. thaliana*, 126–133 Mya; and between *A. thaliana* and *P. tremuloides*, 102–113 Mya. Moreover, gene families in phylogenetic tree-constructing species that had experienced expansion or contraction were identified using CAFÉ v.4.2.1 (De Bie et al., 2006). Genes belonging to particular extended gene families were then functionally analyzed using GO and KEGG enrichment with TBtools (Chen et al., 2023).

### Detecting key candidate functional genes

To find members of the gene families implicated in the pathways leading to terpen biosynthesis, we conducted searches using BLASTP and Hidden Markov Models (HMMs). In particular, we gathered genes that are known to be connected to this *A. thaliana* pathway. Then, BLASTP (e < 1e-5) was performed to discover pathway genes in the genome of *C. longepaniculata* using these genes as query sequences. HMM data for each gene family’s conserved protein domain were downloaded in the interim using the Pfam website (https://pfam.xfam.org/). These HMM files were used in batch searches, along with HMMER v3.2.1 (Johnson et al., 2010). These HMM files served as the basis for batch searches. The BLASTP-identified candidate genes that lacked the matching domain were eliminated. We utilized the PF02458 Pfam domain for BAHD searching and the PF01397 and PF03936 Pfam domains for HMMER searching in order to identify the *TPS* genes. In order to differentiate between distinct subfamilies, phylogenetic trees were created for each gene family’s candidate genes using IQTREE v.2.1.4-beta (Nguyen et al., 2015).

### Transcriptomic analysis

We used transcriptome data from the leaf to do RNA-seq analysis to look into the expression levels of genes involved in the terpene synthesis pathway. We first performed a raw data quality assessment for transcriptome analysis using FASTQC (https://www.bioinformatics.babraham.ac.uk/projects/fastqc/). Subsequently, quality control was performed using FASTP v0.20.0 to filter out raw reads containing poly-N sequences and low-quality reads (< Q30). We used the high-quality reads obtained from the quality control step for the subsequent expression analysis. After filtering the high-quality RNA-seq reads, we used HISAT2 v.2.1.0 (Kim et al., 2015) to map them to the genome of *C. longepaniculata*. Finally, using StringTie v.1.3.4d (Pertea et al., 2015), we quantified gene expression levels in transcripts per kilobase million (TPM).

## Results

### Genome assembly

In order to create the genome of *C. longepaniculata* (**Figure 1A**), we initially generated 49.43Gb of paired-end reads using the Illumina NovaSeq 6000 (**Supplementary Table S1**). Our 19-mer analysis indicated an estimated genome size of 702 Mb, with a heterozygosity rate of 3.31% (**Supplementary Table S3**). Building on this, we utilized 31.54 Gb of high-quality high-fidelity (HiFi) reads produced on the PacBio platform, achieving 44.91-fold coverage of the *C. longepaniculata* genome (**Supplementary Table S1**). Utilizing the Hifiasm tool, this sequencing effort yielded thirty contigs, totalling 768.10 Mb with an N50 size of 41.12 Mb (**Supplementary Table S4**). To enhance the assembly, we incorporated Hi-C data, employing Hi-C-Pro v2.8.1 to recognize and retain 89.18 Gb of valid interaction paired reads (**Supplementary Table S5**). This integration allowed the assembly to be further consolidated. Ultimately, we anchored 766.69 Mb (99.82%) of the contig sequences into 12 chromosomes (**Figures 1B, C**; **Supplementary Table S6**). The final scaffold N50 was improved to 63.78 Mb, with the longest scaffold measuring 111.09 Mb (**Table 1**; **Supplementary Table S6**). Additionally, a heatmap of chromosome interactions demonstrated the completeness and robustness of the genome assembly (**Figure 1C**).

**Table 1:** Assembly and annotation features from the *Camphora longepaniculata* assembly.

Type	Statistics
Assembly size (bp)	768,095,601
Number of scaffolds	18
Scaffold N50 size (bp)	63,783,332
Number of contigs	33
Contig N50 size (bp)	35,809,667
Number of chromosomes	12
Ordered and oriented genome size (bp) and percentage (%)	766,694,454 99.82
Repeat region size (bp) and percentage (%)	463,826,005 60.39
GC content (%)	38.76
Number of protein-coding genes	39,173
Functional annotated genes (%)	93.51

### Prediction and functional annotation of protein-coding genes

A combination of homology-based, reference-guided transcriptome assembly, and ab initio gene approaches were used to predict gene models in the *C. longepaniculata* assembly. The consensus gene set was then generated by combining the gene prediction results with EVM software. To improve the quality of gene prediction, genes with transposable elements and miscoded genes were eliminated. In the end, 39,173 genes made up the final gene list that we acquired. These protein-coding genes had an average gene length of 7,152.40 bp and an average CDS length of 1,146.30 bp (**Supplementary Tables S7, S8**). There were 4.38 exons on average per gene, with an average exon length of 261.80 bp and intron length of 1608.33 bp (**Supplementary Table S8**).

### Annotation of repeat and noncoding RNAs

Approximately sixty percent of the whole-genome assembly consisted of the detected repetitive sequences (463.83 Mb) (**Supplementary Table S9**). The most common repeat types were DNA elements (19.72%) and long terminal repeat (LTR) retrotransposons (31.96%) (**Supplementary Table S9**). LINE (3.19 Mb) made up 0.42% of the whole genome assembly, in contrast. With computed average lengths of 106.89, 74.99, 191.27, and 118.18 bp, respectively, 6,531 miRNAs, 582 transfer RNAs (tRNAs), 3,312 rRNAs, and 634 snRNAs were found (**Supplementary Table S10**).

### Genome quality assessment

With short reads an overall mapping rate of 99.20% was obtained, encompassing 99.92% of the assembly (**Supplementary Table S11**). Moreover, HiFi reads were mapped back using Minimap2 (Li, 2018), resulting in an assembly coverage of 100% and an overall mapping rate of 99.92% (**Supplementary Table S11**). These findings imply that the genetic information provided in our assembly was almost entirely complete. Moreover, SAMtools v1.4 (Li et al., 2009) was used to identify and filter single-nucleotide polymorphisms (SNPs), resulting in the identification of 11,919,258 heterozygous SNPs, 18,760 homozygous SNPs, and 9,182 homozygous INDELs with 5× sequencing depth. The high precision of the assembly (99.996385%) is supported by the low frequencies of homozygous SNPs and INDELs, which together make up 0.002442% and 0.001195% of the assembled genome, respectively. Ultimately, a scatter plot of the sequencing depth vs the GC-content based on 10-kb windows showed that the completed *C. longepaniculata* genome was free of foreign DNA contamination (**Figure 1B**, **Supplementary Figure S1**). Additionally, we used the embryophyta odb10 database (https://busco.ezlab.org/) to perform BUSCO analysis on the data (Simão et al., 2015). Out of 1,614 conserved plant genes, 4.52% of them contained duplicates, yet 99.01% of them had full coverage in the genome, 0.43% were fragmented, and only 0.56% were missing (**Supplementary Table S12**). These results clearly show that the genome assembly of *C. longepaniculata* we have produced is of excellent quality and has the potential to be useful for future research. The completeness of these anticipated genes was further evaluated using BUSCO analysis, yielding a BUSCO score of 93.31% (single = 89.34%, duplicated = 3.97%, fragmented = 4.96%, missing = 1.73%, genes = 1,614; **Supplementary Table S13**). Furthermore, functions for 65.80% (25,777), 35.86% (14,049), 71.29% (27,928), 36.48% (14,290), 92.78% (36,345), and 93.26% (36,533) of the genes were found by searching the Swiss-Prot, KEGG, COG, GO, TrEMBL, and NR databases, respectively (**Supplementary Table S14**). A total of 36,632 protein-coding genes, or 93.51% of them, had their conserved functional motifs or functional terms effectively annotated (**Supplementary Table S14**). These findings suggest that,the *C. longepaniculata* genome’s annotated gene set is rather comprehensive.

### Phylogeny and evolution of gene families

**Figure 2:**
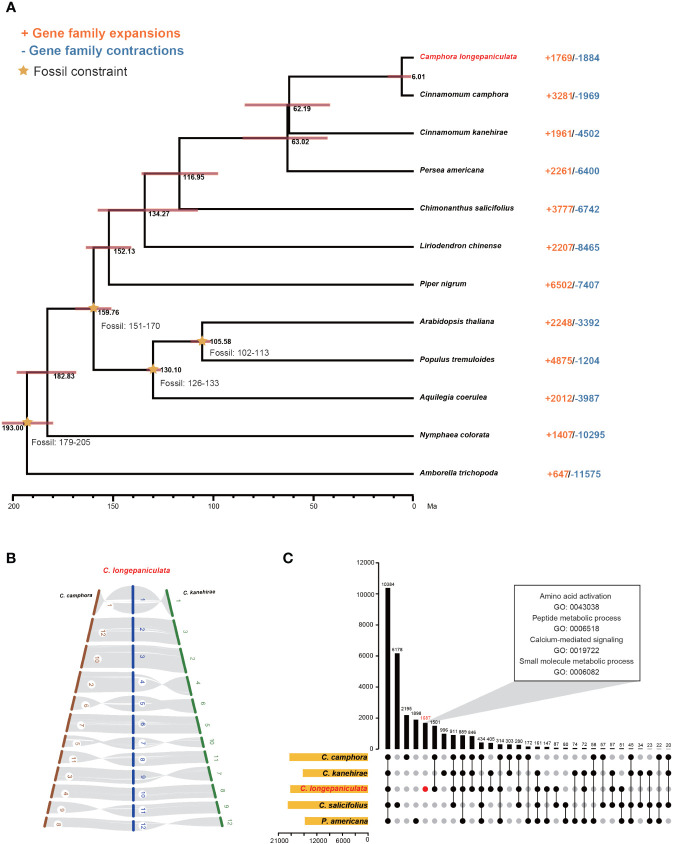
Comparative genomic, Co-linearity and Gene family sharing analysis, **(A)** Comparative genomic analysis of *Camphora longepaniculata* and other plant species. Annotated differentiation time, a 95% confidence interval for differentiation time, and the expansion or contraction of gene families in each species. **(B)** Co-linearity of *Cinnamomum camphora*, *Cinnamomum kanehirae*, and *Camphora longepaniculata*. **(C)** Gene family sharing in *C. longepaniculata*, *C. camphora*, *C. kanehirae*, *C. salicifolius*, and *P. americana*.

As described in the Materials and Methods section, 288 single-copy orthologous genes were discovered by analyzing the genomes of 12 different species. *C. longepaniculata* and *C. camphora* were found to have a closer relationship based on phylogenetic analyses of these genes. *C. longepaniculata* split from *C. camphora* and *C. kanehirae* between 6.01 million and 62.19 million years ago, according to projected divergence times (**Figure 2A**). Furthermore, the collinearity findings revealed a high level of collinearity among the three species (**Figure 2B**), and *C. longepaniculata* was discovered to have a WGD event shared by *Lauraceae* species. In addition, *C. longepaniculata* shared 10,384 gene families with four other species (*C. camphora*, *C. kanehirae*, *C. salicifolius*, and *P. americana*) and had 1,687 unique gene families (**Figure 2C**). We further investigated structural variations (SVs) among *C. longepaniculata*, *C. camphra*, and *C. kanehhirae*. We annotated a total of 695,731 SVs (including small insertions and deletions) between *C. longepaniculata* and *C. camphra*, and 801,107 SVs between *C. longepaniculata* and *C. kanehhirae* after whole-genome alignment using minimap2 and SV calling using SyRI (**Supplementary Figure S3**). After filtering, we focused on SVs longer than 10kb, resulting in the retention of 949 (between *C. longepaniculata* and *C. camphora*) and 701 (between *C. longepaniculata* and *C. kanehirae*) SVs, respectively. The analysis of functional enrichment of genes within 10kb of SV breakpoints showed significant enrichment in terms of terpene synthase activity (GO:0010333), terpenoid biosynthetic process (GO:0016114), monoterpene metabolic process (GO:0016098), and chloroplast RNA processing (GO:0031425), among others. This suggests that post-WGD diploidization may play a part in improving the terpenoid biosynthesis pathway (**Supplementary Table S15**).

After a thorough evaluation of gene families in each of the 12 species, it was determined that 1,769 extended families and 1,884 contracted families are included in the genome of *C. longepaniculata* (**Figure 2A**). Statistical analysis revealed that 584 expanded and 497 contracted gene families were statistically significant (P < 0.05; **Supplementary Tables S16**, **S17**). These gene families were clarified by the Gene Ontology (GO) enrichment study. Significantly, gene families linked to the following were found to have expanded: “transferase activity (GO: 0016740)”, “catalytic activity (GO: 0003824)”, “response to oomycetes (GO: 0002239)”, “regulation of hydrogen peroxide metabolic process (GO: 0010310)”, and “secondary metabolic process (GO: 0019748)” (**Supplementary Table S16**). These gene families may help to explain the high levels of oil content and environmental resistance in *C. longepaniculata*. The gene families for “vesicle coating (GO: 0006901), Golgi vesicle transport (GO: 0048193), response to hydroperoxide (GO: 0033194), and channel activity (GO: 0015267)” on the other hand, showed shrinkage (**Supplementary Table S17**). Moreover, processes like “amino acid activation (GO: 0043038),” “peptide metabolic process (GO: 0006518),” “calcium-mediated signaling (GO: 0019722),” “organic acid metabolic process (GO: 0006082),” and “small molecule metabolic process (GO: 0044281)” were identified by GO enrichment analysis focused on *C. longepaniculata* specific gene families. These processes are all crucial to the process of oil biosynthesis (**Figure 2C**; **Supplementary Table S18**).

### Analysis of terpene biosynthesis and related genes

**Figure 3:**
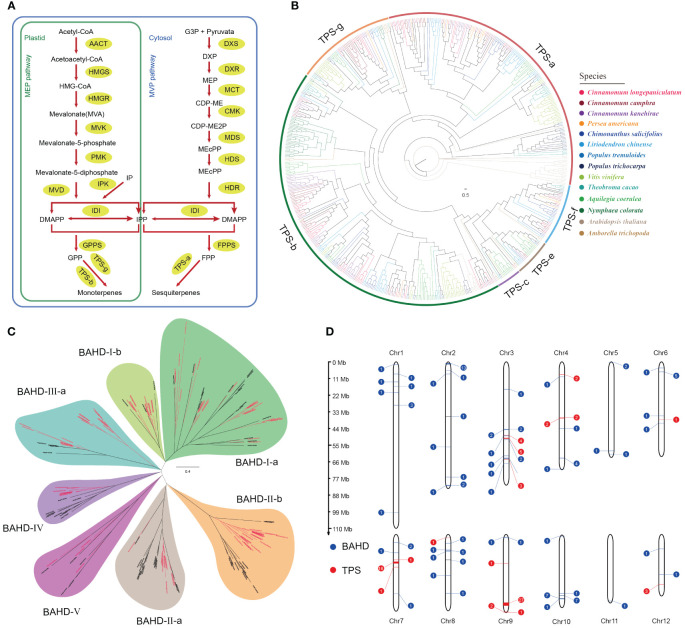
Genomic insights into terpenoid biosynthesis and distribution in *Camphora longepaniculata*. **(A)** MEP and MVP pathway for the biosynthesis of terpenoid. **(B)** Phylogenetic analysis of *TPS*s from *Camphora longepaniculata* and other plant species. **(C)** Phylogenetic analysis of *BAHD*s from *Camphora longepaniculata* and *Arabidopsis thaliana.* **(D)** Distribution of *TPS* and *BAHD* genes on chromosomes in *Camphora longepaniculata*.

Due to the high concentration of terpenoids (volatile organic compounds) in the leaf essential oil of *C. longepaniculata* and their role as important sources of plant fragrance, we focused on the terpene biosynthesis pathway genes in the *C. longepaniculata* genome. Terpenoids (monoterpenes, sesquiterpenes and iridoids) are usually synthesized via the MVA and MEP pathways (**Figure 3A**). We identified a total of 37 relevant genes from these two pathways in the *C. longepaniculata* genome (**Supplementary Table S19**). The results showed that there was no significant difference in the copy number of these genes in the Lauraceae species overall. However, the MVD genes, which encodes diphosphomevalonate decarboxylase, showed significant expansion compared to other species, reaching up to 5 copies in *C. longepaniculata* (**Supplementary Table S19**). These genes are responsible for catalyzing the conversion of mevalonate-5-diphosphate to isopentenyl diphosphate (IPP), which is one of the important precursors for synthesizing various terpenoids. This reaction step links the early steps of terpene biosynthesis with the subsequent conversion steps. The expansion of the MVP gene family in the genome of *C. longepaniculata* may be of significance for its terpenoid biosynthesis.

On the other hand, 1,8-Cineole, α-terpineol, and γ-terpinene are important constituents of the leaf essential oil in *C. longepaniculata*, and these three compounds all belong to the class of monoterpenes. Subsequently, we focused on terpene synthase (TPS), which is crucial in the biosynthesis of terpenes. It catalyzes a variety reactions in the MVA and MEP pathways to produce the basic skeletons of terpenoids. We identified 74 genes encoding terpene synthase in *C. longepaniculata* (**Supplementary Table S20**). Notably, the *TPS* gene family in *Lauraceae* species significantly expanded compared to other selected species (*C. longepaniculata*: 74; *C. camphora*: 75; *C. kanehirae*: 88; **Supplementary Table S20**). More tree-based study of these *TPS* genes showed that the *TPS-b* and *TPS-g* subfamilies have grown in Lauraceae species (**Figure 3B**; **Supplementary Table S20**). Members of these two subfamilies mainly synthesize non-cyclic monoterpenes, sesquiterpenes, and diterpenes. This partially clarifies the significant contribution of the *TPS-b* and *TPS-g* gene families’ expansion to the accumulation of monoterpenes in *C. longepaniculata*’s leaf essential oil. BAHD acyltransferases, which participate in the synthesis of various flavors and fragrances in plants, generally transform terpenes into esters. In the genome of *C. longepaniculata*, we identified 98 genes that belong to the *BAHD* gene family. Phylogenetic analysis showed that the growth of different subfamilies of the *BAHD* gene family was significantly different between species (**Figure 3C**; **Supplementary Table S21**). We observed expansion trend in several subfamilies of Lauraceae species, including *BAHD-I-b*, *BAHD-II-b*, and *BAHD-III-a*. Specifically, for *C. longepaniculata*, the number of genes in the *BAHD-II-b* subfamily reached up to 19 (**Supplementary Table S21**). Also, the *BAHD-II-b* and *BAHD-III-a* subfamilies of *C. longepaniculata* formed a separate sub-branch compared to the reference sequence of *A. thaliana*. This suggests that the lineage specificity of the *BAHD-II-b* and *BAHD-III-a* genes may have different functions, which may help create the unique smell of *C. longepaniculata.* Observation of the chromosomal locations of *TPS* and *BAHD* gene families revealed that, similar to previous results in other plants (Xu et al., 2022), many *TPS* genes in *C. longepaniculata* exhibited tandem repeats. Specifically, these *TPS* genes formed gene clusters on chromosomes 3, 4, 7, and 9, showing a distinct clustered distribution pattern. On chromosomes 7 and 9, we found two large gene clusters, one with 18 genes and the other with 27 genes. This suggests that tandem duplication events recently created these genes (**Figure 3D**). At the same time, we observed several gene clusters on chromosomes 2 and 10 for the distribution of *BAHD* genes their overall distribution did not follow the same clustered pattern as *TPS*.

In comparison, our findings show that, like *C. camphora* and *C. kanehirae*, *C. longepaniculata* has similar gene positions and good collinearity on the same chromosomes (**Supplementary Figure S3**). Tandem repeats, such as *MVD1* and *MVD2* in *C. longepaniculata*, as well as *MVD3*, *MVD4*, and *MVD5* (**Supplementary Figure S3**), account for the majority of the differences in gene copy numbers between these genomes. Furthermore, structural arrangements lead to gene duplications, as seen in the cases of *FPPS2* and *FPPS3*, which most likely resulted from chromosome segmental duplication events. Notably, an inversion on chromosome 9 reconfigured the *TPS* gene cluster, resulting in a more densely packed arrangement of these genes, which was also found in the *C. camphora* genome (**Supplementary Figure S3**). This data further demonstrates that pathway genes are highly expressed, with at least one gene from each family (**Supplementary Figure S4**).

## Discussion

*Camphora longepaniculata*’s chromosome-level genome assembly represents a significant step forward in Lauraceae genomic investigations. The assembly has the longest contig or scaffold N50 values reported for this family—41.11 Mb and 63.78 Mb, respectively—and an impressive genome completeness of 99.01% as assessed by BUSCO results (**Table 1**; **Supplementary Tables S3**, **S7**; Chaw et al., 2019; Han et al., 2022; Li et al., 2022; Shen et al., 2022; Xiong et al., 2022).With its high-quality metrics, it establishes a new benchmark. Besides, our mapping Illumina short reads, SNP validation, and Poisson distribution analysis (**Supplementary Figure S1**, **Supplementary Table S11**) collectively affirmed the reliability, accuracy, and completeness of *C. longepaniculata* genome assembly. This validation is critical for demonstrating our genomic data’s dependability in future biological studies. We need this high-quality genomic data to learn more about the genetic structure and metabolic abilities of *C. longepaniculata*, especially how it makes terpenoids, which are important parts of the leaf essential oil that have big economic and environmental values (Li et al., 2012, 2014). Our findings greatly increase our understanding of the *Camphora* genus, which is commonly mistaken for *Cinnamomum* in the Lauraceae family. New phylogenetic research and molecular data show that *Cinnamomum* is polyphyletic. *Camphora* is now a separate genus, containing 18 species that form a monophyletic group, according to Rohwer et al. (2019) and Yang et al. (2022). This differentiation is critical because *Camphora* species, found across Asia’s tropical and subtropical climates, have specific evergreen features that differ significantly from those of *Cinnamomum*. It’s important to know the differences between *Camphora* and other *Cinnamomum* species by looking at their genetic traits. For example, *Camphora* has alternating, pinnately veined leaves and fruiting tepals that don’t stay in place, while some *Cinnamomum* species have opposite, triplivined leaves and persistent tepals. Our genomic study backs up these results by showing that *C. longepaniculata* and *C. camphora* share more gene families and fewer structural variants (SVs) than *C. kanehirae* (**Figure 2**; **Supplementary Figure S3**). This genetic similarity supports taxonomic distinction and indicates evolutionary divergences tailored to specific ecological niches. Furthermore, our findings are consistent with morphological observations published by Gang et al. (2021) and Abeysinghe et al. (2020), giving a solid foundation for understanding the evolutionary processes of these taxa. Our comparative genomic analyses, which include divergence times and gene family compositions, not only show that *Camphora* has a unique genetic lineage, but they also help us learn more about its evolutionary history and how it has changed over time (**Figure 2**). This genomic clarity enables a more in-depth examination of *Camphora*’s distinctive features, as well as their ecological and evolutionary consequences, laying the groundwork for future research on biodiversity conservation and the sustainable usage of these species. The genetic divergence between *Camphora* and *Cinnamomum* emphasizes the necessity of proper taxonomic classifications in advancing our understanding of plant biology and informing conservation initiatives. Furthermore, the genome data from *C. longepaniculata* sheds light on magnoliid evolution, providing a more complete evolutionary context within the angiosperms. This closes a major gap in our evolutionary knowledge.

The identification of a robust ensemble of genes participating in the MVA and MEP pathways, particularly the expansion of the MVD genes, demonstrates a genetic propensity for increased terpenoid synthesis (**Figure 3**; **Supplementary Table S19**). This enlargement, which is suggestive of evolutionary adaptation, increases the plant’s ability to synthesize terpenoids. These chemicals are important for the plant’s survival and ability to interact with its environment because they help protect it and attract pollinators (Nerg et al., 2004; Della Rocca et al., 2020). Surprisingly, the discovery of 74 *TPS* genes—a substantially higher number than observed in other species—demonstrates the plant’s particular capacity for terpene synthesis (**Supplementary Table S20**). TPS enzymes are known for accelerating the production of the basic carbon skeletons of terpenoids, hence their quantity and diversity indicate the plant’s ability to synthesize a wide range of terpenoid molecules. The growth of specific TPS subfamilies, particularly TPS-b and TPS-g, which are primarily involved in the synthesis of non-cyclic monoterpenes, sesquiterpenes, and diterpenes, may contribute to the high concentration of these chemicals in *C. longepaniculata* essential oils (Chen et al., 2011). This association not only confirms the functional relevance of these gene expansions, but also shows their potential contribution to the species’ specific aromatic qualities. Furthermore, the expansion of the BAHD acyltransferase gene family in *C. longepaniculata*, as evidenced by lineage-specific expansions in subfamilies BAHD-II-b and BAHD-III-a, indicates evolutionary adaptation for more complex terpenoid modifications into aroma compounds (**Figure 3C**; **Supplementary Table S21**; Srivastava and Sangwan, 2012). This increase is consistent with the family’s recognized role in esterification, which is essential for producing varied flavors and scents and so contributes to the species’ distinct aromatic properties (Souleyre et al., 2005; Cumplido-Laso et al., 2012). The chromosomal mapping of *TPS* and *BAHD* genes improves our understanding of the genomic architecture that underpins terpenoid biosynthesis. The presence of large gene clusters, notably *TPS* genes on chromosomes 7 and 9, suggests recent tandem duplication events, which may be promoting the diversity of terpene biosynthesis enzymes in *C. longepaniculata* (**Figure 3D**). This genomic architecture is consistent with observations from *C. camphora*, indicating a shared evolutionary approach within the genus (Shen et al., 2022). This pattern of gene duplication and clustering is prevalent in plant genomes and is frequently linked to the rapid evolution of gene families that respond to ecological and environmental stressors (Yang et al., 2021; Lei et al., 2024).

The identification of SVs associated with terpenoid synthesis genes, notably inside the greatest tandem repeat sections of *TPS* genes on chromosome 9 of the *C. longepaniculata* genome, provides deep insights into the plant’s evolutionary biology (**Figure 3D**; **Supplementary Figure S3**). These findings imply that SVs play a substantial role in the diversification and specialization of terpenoid biosynthesis pathways, emphasizing their importance as evolutionary tools that allow *C. longepaniculata* to tune chemical outputs to environmental stresses. The link between genetic rearrangements and metabolic pathway specialization improves our understanding of terpenoid production’s molecular dynamics and identifies prospective targets for biotechnological improvements targeted at improving terpenoid yield and variety. Our study highlights critical targets for genetic engineering to increase the production and diversity of terpenoids, which have significant commercial and ecological significance. Using full genomic knowledge could transform approaches for analyzing gene activity within organisms, hastening developments in agriculture and pharmaceuticals. *C. longepaniculata*’s unique biochemical capabilities provide opportunities to generate new products and improve existing ones by using its genetic resources. This genomic research sets the path for future studies that use *C. longepaniculata*’s inherent genetic variety to potentially increase terpenoid production, contributing to the fields of natural product chemistry and sustainable resource management. It emphasizes the significance of combining genomic data with ecological and evolutionary insights in order to fully realize plant species’ potential in a changing global context.

## Conclusion

Our study presents a chromosome-level genome assembly of *C. longepaniculata* using HiFi sequencing, which was supplemented with short-read and Hi-C sequencing. *C. longepaniculata* genomic analysis has provided valuable insights into its genetic composition and functional characteristics. We identified key patterns of gene family sharing and divergence using comparative genomics with 11 other species, shedding light on the relationships between *C. longepaniculata* and closely related species like *C. camphora*. The estimated divergence times, collinearity results, and evidence of Whole Genome Duplication (WGD) provide a complete picture of the species’ evolutionary history. The fact that the *TPS* and *BAHD* gene families have grown so much in the genome shows how important secondary metabolite pathways have been for evolution as ways for *Camphora* species to adapt. This is in line with ecological and evolutionary theories that say secondary metabolites are very important for plants to adapt and survive. This genomic characterization of *C. longepaniculata* lays the groundwork for more biochemical and ecological studies that will look into how these genomic features affect function. This could pave the way for the biotechnological use of these natural products.

## Data availability statement

The data presented in the study are deposited in the NCBI repository, accession number PRJNA1068955. The genome assembly file, all the annotation files, and source data for phylogenetic and population analyses are available at Figshare (https://figshare.com/s/ff6a0f810527f61ef63c). All other data are available from the corresponding authors on reasonable request.

## Author contributions

KY: Conceptualization, Formal analysis, Funding acquisition, Methodology, Project administration, Resources, Software, Writing – original draft, Writing – review & editing. HZ: Conceptualization, Data curation, Investigation, Methodology, Visualization, Writing – review & editing. GC: Data curation, Validation, Writing – review & editing. LM: Data curation, Formal analysis, Writing – review & editing. JL: Methodology, Validation, Writing – review & editing. JZ: Investigation, Software, Writing – review & editing. SL: Formal analysis, Validation, Writing – review & editing. YW: Data curation, Validation, Writing – review & editing. RF: Formal analysis, Investigation, Writing – review & editing. SS: Formal analysis, Software, Writing – review & editing. ME: Formal analysis, Software, Writing – review & editing. RH: Formal analysis, Software, Writing – review & editing. QW: Conceptualization, Methodology, Validation, Writing – original draft, Writing – review & editing. AE-S: Conceptualization, Methodology, Writing – original draft, Writing – review & editing. DR: Conceptualization, Methodology, Validation, Writing – original draft, Writing – review & editing.

## Supplementary material

Supplementary Figure 1 Scatter plot illustrating the sequencing depth versus the GC-content based on 10-kilobase (kb) windows.

Supplementary Figure 2 Intra-genomic comparison within *Camphora longepaniculata.*

Supplementary Figure 3 SVs identified between *Camphora longepaniculata* and *Cinnamomum camphora* (top) and between *Camphora longepaniculata* and *Cinnamomum kanehirae* (bottom) across the 12 chromosomes.

Supplementary Figure 4 Expression level of pathway genes.

## References

[B1] Abeysinghe P. D. Bandaranayake P. C. Pathirana R. (2020). Botany of endemic Cinnamomum species of Sri Lanka. Cinnamon: Botany Agronomy Chem. Ind. Appl., 85–118. doi: 10.1007/978-3-030-54426-3_4

[B2] Altschul S. F. Madden T. L. Schäffer A. A. Zhang J. Zhang Z. Miller W.. (1997). Gapped BLAST and PSI-BLAST: a new generation of protein database search programs. Nucleic Acids Res. 25, 3389–3402. doi: 10.1093/nar/25.17.3389 9254694 PMC146917

[B3] Bairoch A. Apweiler R. (2000). The SWISS-PROT protein sequence database and its supplement TrEMBL in 2000. Nucleic Acids Res. 28, 45–48. doi: 10.1093/nar/28.1.45 10592178 PMC102476

[B4] Bao W. Kojima K. K. Kohany O. (2015). Repbase Update, a database of repetitive elements in eukaryotic genomes. Mobile DNA 6, 1–6. doi: 10.1186/s13100-015-0041-9 PMC4455052 26045719

[B5] Belton J.-M. McCord R. P. Gibcus J. H. Naumova N. Zhan Y. Dekker J. (2012). Hi–C: a comprehensive technique to capture the conformation of genomes. Methods 58, 268–276. doi: 10.1016/j.ymeth.2012.05.001 22652625 PMC3874846

[B6] Benson G. (1999). Tandem repeats finder: a program to analyze DNA sequences. Nucleic Acids Res. 27, 573–580. doi: 10.1093/nar/27.2.573 9862982 PMC148217

[B7] Boutanaev A. M. Moses T. Zi J. Nelson D. R. Mugford S. T. Peters R. J.. (2015). Investigation of terpene diversification across multiple sequenced plant genomes. Proc. Natl. Acad. Sci. U.S.A. 112, E81–E88. doi: 10.1073/pnas.1419547112 25502595 PMC4291660

[B8] Chaw S.-M. Liu Y.-C. Wu Y.-W. Wang H.-Y. Lin C.-Y. I. Wu C.-S.. (2019). Stout camphor tree genome fills gaps in understanding of flowering plant genome evolution. Nat. Plants 5, 63–73. doi: 10.1038/s41477-018-0337-0 30626928 PMC6784883

[B9] Chen C. Wu Y. Li J. Wang X. Zeng Z. Xu J.. (2023). TBtools-II: A “one for all, all for one“ bioinformatics platform for biological big-data mining. Mol. Plant 16, 1733–1742. doi: 10.1016/j.molp.2023.09.010 37740491

[B10] Chen F. Tholl D. Bohlmann J. Pichersky E. (2011). The family of terpene synthases in plants: a mid-size family of genes for specialized metabolism that is highly diversified throughout the kingdom. Plant J. 66, 212–229. doi: 10.1111/j.1365-313X.2011.04520.x 21443633

[B11] Chen S. Zhou Y. Chen Y. Gu J. (2018). fastp: an ultra-fast all-in-one FASTQ preprocessor. Bioinformatics 34, i884–i890. doi: 10.1093/bioinformatics/bty560 30423086 PMC6129281

[B12] Cheng H. Concepcion G. T. Feng X. Zhang H. Li H. (2021). Haplotype-resolved *de novo* assembly using phased assembly graphs with hifiasm. Nat. Methods 18, 170–175. doi: 10.1038/s41592-020-01056-5 33526886 PMC7961889

[B13] Cumplido-Laso G. Medina-Puche L. Moyano E. Hoffmann T. Sinz Q. Ring L.. (2012). The fruit ripening-related gene FaAAT2 encodes an acyl transferase involved in strawberry aroma biogenesis. J. Exp. Bot. 63, 4275–4290. doi: 10.1093/jxb/ers120 22563120

[B14] De Bie T. Cristianini N. Demuth J. P. Hahn M. W. (2006). CAFE: a computational tool for the study of gene family evolution. Bioinformatics 22, 1269–1271. doi: 10.1093/bioinformatics/btl097 16543274

[B15] Della Rocca G. Danti R. Hernando C. Guijarro M. Michelozzi M. Carrillo C.. (2020). Terpenoid accumulation links plant health and flammability in the cypress-bark canker pathosystem. Forests 11, 651. doi: 10.3390/f11060651

[B16] Dobin A. Davis C. A. Schlesinger F. Drenkow J. Zaleski C. Jha S.. (2013). STAR: ultrafast universal RNA-seq aligner. Bioinformatics 29, 15–21. doi: 10.1093/bioinformatics/bts635 23104886 PMC3530905

[B17] Dudchenko O. Batra S. S. Omer A. D. Nyquist S. K. Hoeger M. Durand N. C.. (2017). *De novo* assembly of the Aedes aEgypti genome using Hi-C yields chromosome-length scaffolds. Science 356, 92–95. doi: 10.1126/science.aal3327 28336562 PMC5635820

[B18] Emms D. M. Kelly S. (2019). OrthoFinder: phylogenetic orthology inference for comparative genomics. Genome Biol. 20, 1–14. doi: 10.1186/s13059-019-1832-y 31727128 PMC6857279

[B19] Flynn J. M. Hubley R. Goubert C. Rosen J. Clark A. G. Feschotte C.. (2020). RepeatModeler2 for automated genomic discovery of transposable element families. Proc. Natl. Acad. Sci. 117, 9451–9457. doi: 10.1073/pnas.1921046117 32300014 PMC7196820

[B20] Galperin M. Y. Makarova K. S. Wolf Y. I. Koonin E. V. (2015). Expanded microbial genome coverage and improved protein family annotation in the COG database. Nucleic Acids Res. 43, D261–D269. doi: 10.1093/nar/gku1223 25428365 PMC4383993

[B21] Gang Z. Liu B. Rohwer J. G. Ferguson D. K. Yang Y. (2021). Leaf epidermal micromorphology defining the clades in Cinnamomum (Lauraceae). PhytoKeys 182, 125. doi: 10.3897/phytokeys.182.67289 34720625 PMC8516828

[B22] Haas B. J. Salzberg S. L. Zhu W. Pertea M. Allen J. E. Orvis J. (2008). Automated eukaryotic gene structure annotation using EVidenceModeler and the Program to Assemble Spliced Alignments. Genome Biol. 9, R7. doi: 10.1186/gb-2008-9-1-r7 18190707 PMC2395244

[B23] Han X. Zhang J. Han S. Chong S. Meng G. SOng M.. (2022). The chromosome-scale genome of *Phoebe bournei* reveals contrasting fates of terpene synthase (TPS)-a and TPS-b subfamilies. Plant Commun. 3, 100410. doi: 10.1016/j.xplc.2022.100410 35841151 PMC9700126

[B24] Hu W. Gao H. Jiang X. Yang H. (2012). Analysis on constituents and contents in leaf essential oil from three chemical types of Cinnamum camphora. J. Cent. South Univ. Forestry Technol. 32, 186–194.

[B25] Huang J.-F. Li L. van der Werff H. Li H.-W. Rohwer J. G. Crayn D. M.. (2016). Origins and evolution of cinnamon and camphor: A phylogenetic and historical biogeographical analysis of the Cinnamomum group (Lauraceae). Mol. Phylogenet. Evol. 96, 33–44. doi: 10.1016/j.ympev.2015.12.007 26718058

[B26] Johnson L. S. Eddy S. R. Portugaly E. (2010). Hidden Markov model speed heuristic and iterative HMM search procedure. BMC Bioinf. 11, 431. doi: 10.1186/1471-2105-11-431 PMC2931519 20718988

[B27] Katoh K. Standley D. M. (2013). MAFFT multiple sequence alignment software version 7: improvements in performance and usability. Mol. Biol. Evol. 30, 772–780. doi: 10.1093/molbev/mst010 23329690 PMC3603318

[B28] Keilwagen J. Wenk M. Erickson J. L. Schattat M. H. Grau J. Hartung F. (2016). Using intron position conservation for homology-based gene prediction. Nucleic Acids Res. 44, e89. doi: 10.1093/nar/gkw092 26893356 PMC4872089

[B29] Kim D. Langmead B. Salzberg S. L. (2015). HISAT: a fast spliced aligner with low memory requirements. Nat. Methods 12, 357–360. doi: 10.1038/nmeth.3317 25751142 PMC4655817

[B30] Lagesen K. Hallin P. Rødland E. A. Staerfeldt H. H. Rognes T. Ussery D. W. (2007). RNAmmer: consistent and rapid annotation of ribosomal RNA genes. Nucleic Acids Res. 35 (9), 3100–3108. doi: 10.1093/nar/gkm160 17452365 PMC1888812

[B31] Lei W. Zhu H. Cao M. Zhang F. Lai Q. Lu S.. (2024). From genomics to metabolomics: Deciphering sanguinarine biosynthesis in *Dicranostigma leptopodum* . Int. J. Biol. Macromol. 257, 128727. doi: 10.1016/j.ijbiomac.2023.128727 38092109

[B32] Li H. (2018). Minimap2: pairwise alignment for nucleotide sequences. Bioinformatics 34 (18), 3094–3100. doi: 10.1093/bioinformatics/bty191 29750242 PMC6137996

[B33] Li H. Durbin R. (2010). Fast and accurate long-read alignment with Burrows-Wheeler transform. Bioinformatics 26, 589–595. doi: 10.1093/bioinformatics/btp698 20080505 PMC2828108

[B34] Li H. Handsaker B. Wysoker A. 1000 Genome Project Data Processing Subgroup (2009). The sequence alignment/map format and SAMtools. Bioinformatics 25, 2078–2079. doi: 10.1093/bioinformatics/btp352 19505943 PMC2723002

[B35] Li F. Huang S. Mei Y. Wu B. Hou Z. Zhan P.. (2022). Genome assembly provided new insights into the *Cinnamomum burmannii* evolution and D-borneol biosynthesis differences between chemotypes. Ind. Crops Prod. 186, 115181. doi: 10.1016/j.indcrop.2022.115181

[B36] Li L. Li Z.-W. Yin Z.-Q. Wei Q. Jia R.-Y. Zhou L.-J.. (2014). Antibacterial activity of leaf essential oil and its constituents from *Cinnamomum longepaniculatum* . Int. J. Clin. Exp. Med. 7, 1721. 25126170 PMC4132134

[B37] Li N. Zu Y. Wang W. (2012). Antibacterial and antioxidant of celery seed essential oil. Chin. Condiment 37, 28–30.

[B38] Liu B. Shi Y. Yuan J. Hu X. Zhang H. Li N.. (2013). Estimation of genomic characteristics by analyzing k-mer frequency in *de novo* genome projects. arXiv.org, arXiv: 1308.2012. vol. 22.

[B39] Lowe T. M. Eddy S. R. (1997). tRNAscan-SE: a program for improved detection of transfer RNA genes in genomic sequence. Nucleic Acids Res. 25, 955–964. doi: 10.1093/nar/25.5.955 9023104 PMC146525

[B40] Marchler-Bauer A. Lu S. Anderson J. B. Chitsaz F. Derbyshire M. K. DeWeese-Scott C.. (2011). CDD: a Conserved Domain Database for the functional annotation of proteins. Nucleic Acids Res. 39, D225–D229. doi: 10.1093/nar/gkq1189 21109532 PMC3013737

[B41] Nawrocki E. P. Eddy S. R. (2013). Infernal 1.1: 100-fold faster RNA homology searches. Bioinformatics 29, 2933–2935. doi: 10.1093/bioinformatics/btt509 24008419 PMC3810854

[B42] Nerg A. M. Heijari J. Noldt U. Viitanen H. Vuorinen M. Kainulainen P.. (2004). Significance of wood terpenoids in the resistance of Scots pine provenances against the old house borer, *Hylotrupes bajulus*, and brown-rot fungus, *Coniophora puteana* . J. Chem. Ecol. 30, 125–141. doi: 10.1023/B:JOEC.0000013186.75496.68 15074661

[B43] Nguyen L.-T. Schmidt H. A. Von Haeseler A. Minh B. Q. (2015). IQ-TREE: a fast and effective stochastic algorithm for estimating maximum-likelihood phylogenies. Mol. Biol. Evol. 32, 268–274. doi: 10.1093/molbev/msu300 25371430 PMC4271533

[B44] Pertea M. Pertea G. M. Antonescu C. M. Chang T.-C. Mendell J. T. Salzberg S. L. (2015). StringTie enables improved reconstruction of a transcriptome from RNA-seq reads. Nat. Biotechnol. 33, 290–295. doi: 10.1038/nbt.3122 25690850 PMC4643835

[B45] Roach M. J. Schmidt S. A. Borneman A. R. (2018). Purge Haplotigs: allelic contig reassignment for third-gen diploid genome assemblies. BMC Bioinf. 19, 460. doi: 10.1186/s12859-018-2485-7 PMC6267036 30497373

[B46] Rohwer J. G. Trofimov D. Mayland-Quellhorst E. Albach D. (2019). Incongruence of morphological determinations and DNA barcode sequences: a case study in Cinnamomum (Lauraceae). Willdenowia 49, 383–400. doi: 10.3372/wi.49.49309

[B47] Shen T. Qi H. Luan X. Xu W. Yu F. Zhong Y.. (2022). The chromosome-level genome sequence of the camphor tree provides insights into Lauraceae evolution and terpene biosynthesis. Plant Biotechnol. J. 20, 244–246. doi: 10.1111/pbi.13749 34783151 PMC8753352

[B48] Simão F. A. Waterhouse R. M. Ioannidis P. Kriventseva E. V. Zdobnov E. M. (2015). BUSCO: assessing genome assembly and annotation completeness with single-copy orthologs. Bioinformatics 31, 3210–3212. doi: 10.1093/bioinformatics/btv351 26059717

[B49] Song K. He M. Yu J. Guan Y. Bai Y. Xin S.. (2019). Characterization of the chloroplast genome of the family Lauraceae plant species, Cinnamomum cassia. Mitochondrial DNA Part B 4, 3906–3907. doi: 10.1080/23802359.2019.1687360 33366245 PMC7707686

[B50] Souleyre E. J. Greenwood D. R. Friel E. N. Karunairetnam S. Newcomb R. D. (2005). An alcohol acyl transferase from apple (cv. Royal Gala), MpAAT1, produces esters involved in apple fruit flavor. FEBS J. 272, 3132–3144. doi: 10.1111/j.1742-4658.2005.04732.x 15955071

[B51] Srivastava S. Sangwan R. S. (2012). Analysis of *Artemisia annua* transcriptome for BAHD alcohol acyltransferase genes: identification and diversity of expression in leaf, stem and root. J. Plant Biochem. Biotechnol. 21, S108–S118. doi: 10.1007/s13562-012-0141-2

[B52] Stanke M. Diekhans M. Baertsch R. Haussler D. (2008). Using native and syntenically mapped cDNA alignments to improve *de novo* gene finding. Bioinformatics 24, 637–644. doi: 10.1093/bioinformatics/btn013 18218656

[B53] Suyama M. Torrents D. Bork P. (2006). PAL2NAL: robust conversion of protein sequence alignments into the corresponding codon alignments. Nucleic Acids Res. 34, W609–W612. doi: 10.1093/nar/gkl315 16845082 PMC1538804

[B54] Tarailo-Graovac M. Chen N. (2009). Using RepeatMasker to identify repetitive elements in genomic sequences. Curr. Protoc. Bioinformatics Chapter 4, 4.10.11–14.10.14. doi: 10.1002/0471250953.bi0410s25 19274634

[B55] Urasaki N. Takagi H. Natsume S. Uemura A. Taniai N. Miyagi N.. (2017). Draft genome sequence of bitter gourd (Momordica charantia), a vegetable and medicinal plant in tropical and subtropical regions. DNA Res. 24, 51–58. doi: 10.1093/dnares/dsw047 28028039 PMC5381343

[B56] Wei Q. Tan Y. Li Q. You L. Wang C. Wang Y.. (2016). Effects of fungal endophytes on cell suspension culture of *Cinnamomum longepaniculatum* . Guangxi Zhiwu/Guihaia 36, 923–929.

[B57] Wingett S. Ewels P. Furlan-Magaril M. Nagano T. Schoenfelder S. Fraser P.. (2015). HiCUP: pipeline for mapping and processing Hi-C data. F1000Res. 4, 1310. doi: 10.12688/f1000research.7334.1 26835000 PMC4706059

[B58] Wu D. Zhu P. Wu H. Dai H. (2022). Industry development status and prospect of *Cinnamomum longepaniculatum* . Open Access Library J. 9, 1–10. doi: 10.4236/oalib.1108616

[B59] Xiong B. Zhang L. Xie L. Li L. He X. Niu Y.. (2022). Genome of Lindera glauca provides insights into the evolution of biosynthesis genes for aromatic compounds. iScience 25 (8), 104761. doi: 10.1016/j.isci.2022.104761 35942100 PMC9356283

[B60] Xu S. Ding Y. Sun J. Zhang Z. Wu Z. Yang T.. (2022). A high-quality genome assembly of *Jasminum sambac* provides insight into floral trait formation and Oleaceae genome evolution. Mol. Ecol. Resour. 22, 724–739. doi: 10.1111/1755-0998.13497 34460989

[B61] Xu Z. Wang H. (2007). LTR_FINDER: an efficient tool for the prediction of full-length LTR retrotransposons. Nucleic Acids Res. 35, W265–W268. doi: 10.1093/nar/gkm286 17485477 PMC1933203

[B62] Yan K. Wei Q. Feng R. Zhou W. (2019). Transcriptome analysis of the effects of endophytic fungi on the biosynthesis of essential oils in *Cinnamomum longepaniculatum* . Int. J. Agric. Biol. 21, 1301–1308. doi: 10.12688/f1000research.7334.1

[B63] Yan K. Wei Q. Feng R. Zhou W. Chen F. (2017). Transcriptome analysis of *Cinnamomum longepaniculatum* by high-throughput sequencing. Electronic J. Biotechnol. 28, 58–66. doi: 10.1016/j.ejbt.2017.05.006

[B64] Yang X. Gao S. Guo L. Wang B. Jia Y. Zhou J.. (2021). Three chromosome-scale Papaver genomes reveal punctuated patchwork evolution of the morphinan and noscapine biosynthesis pathway. Nat. Commun. 12, 6030. doi: 10.1038/s41467-021-26330-8 34654815 PMC8521590

[B65] Yang Z. (2007). PAML 4: phylogenetic analysis by maximum likelihood. Mol. Biol. Evol. 24 (8), 1586–1591. doi: 10.1093/molbev/msm088 17483113

[B66] Yang Z. Liu B. Yang Y. Ferguson D. K. (2022). Phylogeny and taxonomy of cinnamomum (Lauraceae). Ecol. Evol. 12, e9378. doi: 10.1002/ece3.9378 36203627 PMC9526118

[B67] Zdobnov E. M. Apweiler R. (2001). InterProScan—an integration platfor for the signature-recognition methods in InterPro. Bioinformatics 17, 847–848. doi: 10.1093/bioinformatics/17.9.847 11590104

[B68] Zhang B. Chen S. Liu J. Yan Y.-B. Chen J. Li D.. (2022). A high-quality haplotype-resolved genome of common Bermudagrass (Cynodon dactylon L.) provides insights into polyploid genome stability and prostrate growth. Front. Plant Sci. 13, 890980. doi: 10.3389/fpls.2022.890980 35548270 PMC9081840

