# Supplementary figures and images for: Chromosome genome assembly of the Camphora longepaniculata (Gamble) with PacBio and Hi-C sequencing data
Source: Front Plant Sci. 2024 Jun 26;15:1372127. doi: 10.3389/fpls.2024.1372127 (PMC11238478; doi:10.3389/fpls.2024.1372127)

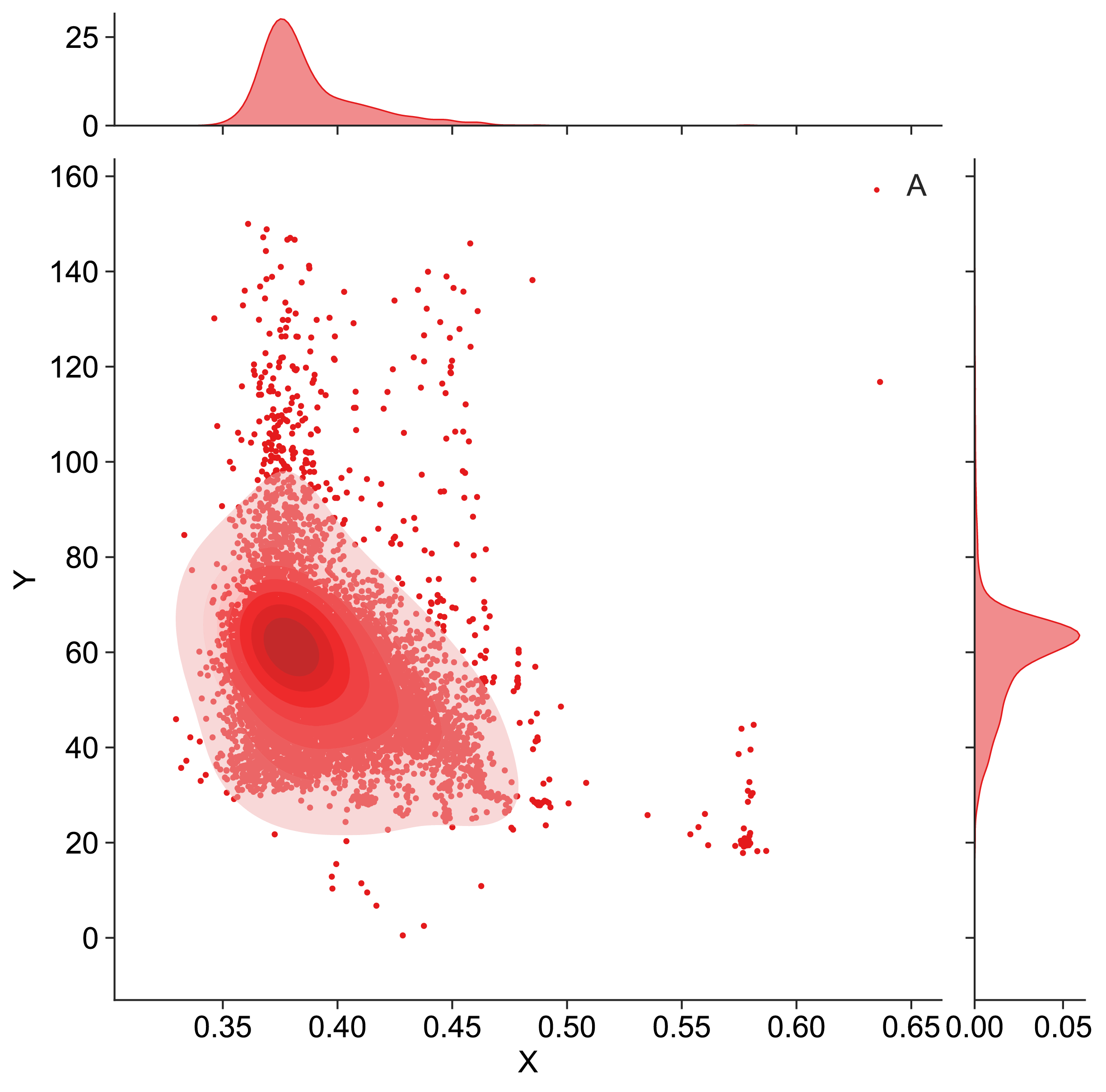

Supplement: Supplementary Figure 1 — Scatter plot illustrating the sequencing depth versus the GC-content based on 10-kilobase (kb) windows. [file Image_1.tif]

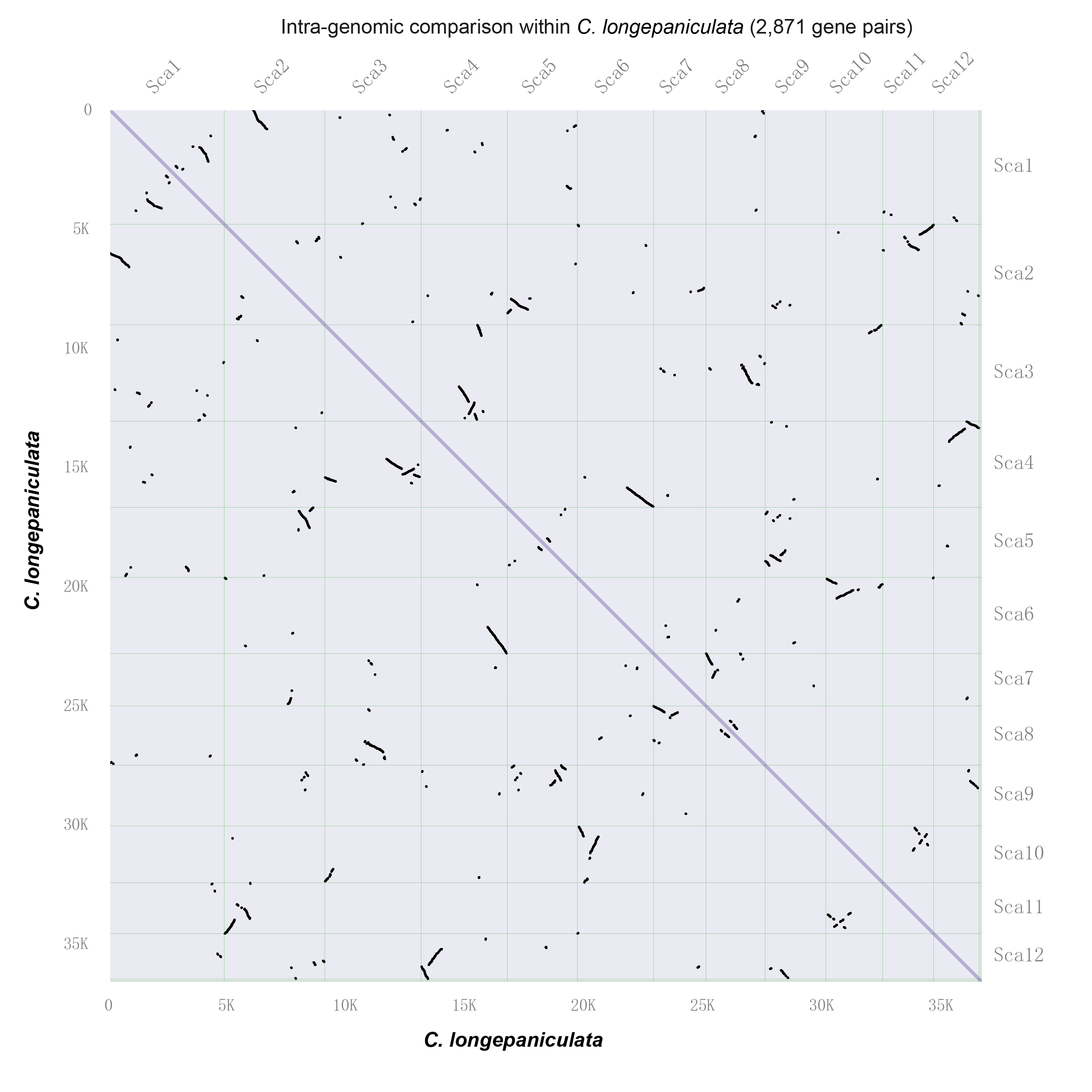

Supplement: Supplementary Figure 2 — Intra-genomic comparison within Camphora longepaniculata. [file Image_2.tif]

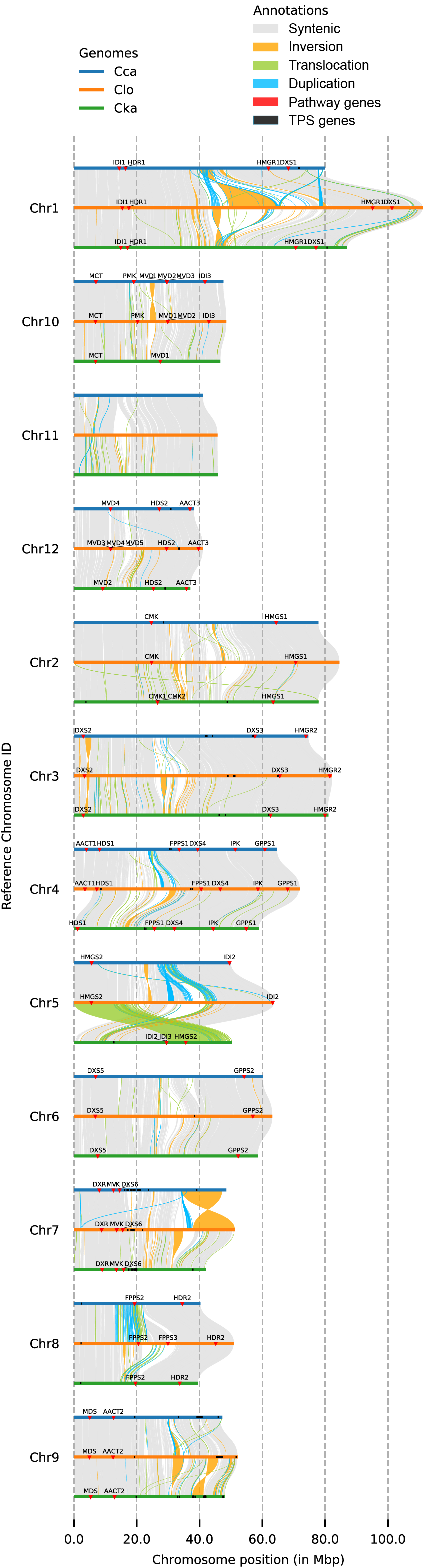

Supplement: Supplementary Figure 3 — SVs identified between Camphora longepaniculata and Cinnamomum camphora (top) and between Camphora longepaniculata and Cinnamomum kanehirae (bottom) across the 12 chromosomes. [file Image_3.tif]

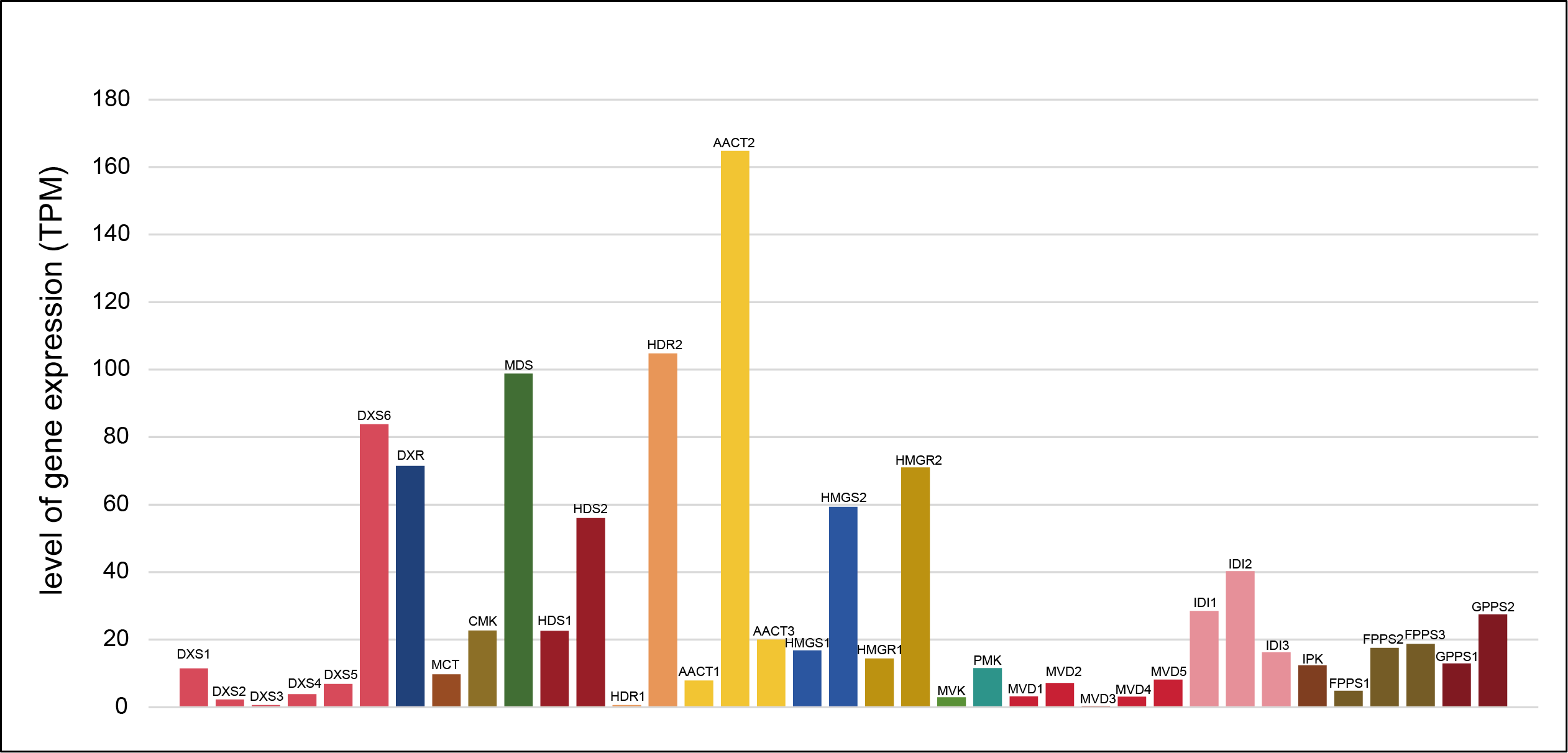

Supplement: Supplementary Figure 4 — Expression level of pathway genes. [file Image_4.tif]
